# Genetic diversity and population structure analysis in cultivated soybean (*Glycine max* [L.] Merr.) using SSR and EST-SSR markers

**DOI:** 10.1371/journal.pone.0286099

**Published:** 2023-05-31

**Authors:** Reena Rani, Ghulam Raza, Muhammad Haseeb Tung, Muhammad Rizwan, Hamza Ashfaq, Hussein Shimelis, Muhammad Khuram Razzaq, Muhammad Arif

**Affiliations:** 1 National Institute for Biotechnology and Genetic Engineering (NIBGE), Faisalabad, Pakistan; 2 Constituent College Pakistan Institute of Engineering and Applied Sciences, Faisalabad, Pakistan; 3 Plant Breeding and Genetics Division, Nuclear Institute of Agriculture (NIA), Tandojam, Pakistan; 4 School of Agricultural, Earth and Environmental Sciences, African Centre for Crop Improvement, University of KwaZulu-Natal, Pietermaritzburg, South Africa; 5 Soybean Research Institute, National Center for Soybean Improvement, Nanjing Agricultural University, Nanjing, China; KGUT: Graduate University of Advanced Technology, ISLAMIC REPUBLIC OF IRAN

## Abstract

Soybean (*Glycine max*) is an important legume that is used to fulfill the need of protein and oil of large number of population across the world. There are large numbers of soybean germplasm present in the USDA germplasm resources. Finding and understanding genetically diverse germplasm is a top priority for crop improvement programs. The current study used 20 functional EST-SSR and 80 SSR markers to characterize 96 soybean accessions from diverse geographic backgrounds. Ninety-six of the 100 markers were polymorphic, with 262 alleles (average 2.79 per locus). The molecular markers had an average polymorphic information content (PIC) value of 0.44, with 28 markers ≥ 0.50. The average major allele frequency was 0.57. The observed heterozygosity of the population ranged from 0–0.184 (average 0.02), while the expected heterozygosity ranged from 0.20–0.73 (average 0.51). The lower value for observed heterozygosity than expected heterozygosity suggests the likelihood of a population structure among the germplasm. The phylogenetic analysis and principal coordinate analysis (PCoA) divided the total population into two major groups (G1 and G2), with G1 comprising most of the USA lines and the Australian and Brazilian lines. Furthermore, the phylogenetic analysis and PCoA divided the USA lines into three major clusters without any specific differentiation, supported by the model-based STRUCTURE analysis. Analysis of molecular variance (AMOVA) showed 94% variation among individuals in the total population, with 2% among the populations. For the USA lines, 93% of the variation occurred among individuals, with only 2% among lines from different US states. Pairwise population distance indicated more similarity between the lines from continental America and Australia (189.371) than Asia (199.518). Overall, the 96 soybean lines had a high degree of genetic diversity.

## Introduction

Soybean, is the world’s fourth most widely grown crop. Its high-quality protein (40%) and vegetable oil (20%) [1, 2], compared to other crops, make it highly desirable for human and animal consumption and as a biofuel [3]. In addition, soybean plays a vital role in nitrogen fixation during crop rotation [4]. At present, Brazil leads all other soybean-growing nations in production and productivity. Indeed, the productivity in other major soybean-growing countries has increased in the last few decades, even though Pakistan remains behind mainly due to stagnant yields. Although there are more than 120.48 million hectares of soybean grown worldwide, but there is a negligible area under soybean cultivation in Pakistan. Agro-ecological conditions of country are favorable for soybean cultivation but still this crop has failed to attain the suitable position in current cropping pattern. The country is spending about two billion US$ on the import of soybean commodities to fulfil local requirements. Apart from the human food products, soybean meal is the main and preferred source of protein for all types of poultry due to good quality of protein and amino acids. Soybean meal is more frequently used in Pakistan’s poultry industry’s feed items. Although agro-ecological conditions of Pakistan favor soybean production, low genetic diversity has hindered the development of new varieties [5–8]. Several studies based on molecular markers and inbreeding coefficient analysis have revealed genetic uniformity in Brazilian soybean cultivars [9, 10]. This limited genetic diversity in elite soybean germplasm indicates that the genes present in current cultivars evolved from a small number of accessions. A more varied genetic background is desirable to protect against unexpected pest and disease outbreaks [11, 12].

For plant breeders, diverse genetic resources increase the chance of developing new and improved cultivars with desired traits [13]. In present, considering the large number of genes predicted to be involved in the control of agronomic traits,.main focus for developing modern cultivars is to locate the best alleles linked to these traits. Presumably, during soybean domestication and introduction in producing regions, a large number of advantageous alleles were lost as a result of genetic bottlenecks. The accessions chosen for a breeding programme must contain and transmit advantageous rare alleles that are lacking in elite germplasm. As a result, understanding the origins of these alleles is crucial. Accessions that are very different from elite genotypes are likely to offer novel alleles for the desired trait. The difficult part is to choose accessions from the available germplasm to use in breeding operations. Therefore, knowledge of the genetic diversity of soybean genotypes would help breeders and geneticists understand the structure of the germplasm to choose parents with greater genetic diversity and accelerate the expansion of the genetic resources [14]. Morphological characterization, biochemical markers, and molecular marker techniques are frequently used to access genetic diversity among and between populations [15]. Morphological and biochemical markers are less reliable than DNA markers due to significant environmental effects [16]. Developing DNA markers is important for understanding the genetic diversity between and within different crop species [17, 18] as they draw attention to variations in the nucleotide sequence between different individuals and are indifferent to environmental variables [19]. Molecular markers such as Random amplified polymorphic DNA (RAPD), Simple- sequence repeats (SSR), expresses sequence tags (EST-SSR), Amplified fragment length polymorphism (AFLP), and Single nucleotide polymorphism (SNP) have been used to identify genetic diversity in soybean germplasm [20–26].SSR markers have been the most used for characterizing genes, analyzing genetic diversity, and mapping genetic linkages. SSR markers are very useful for genotype differentiation, pedigree analysis, assessing genetic distances among genotypes, and variety identification because they are short tandem repeats dispersed uniformly on the entire genome with high polymorphic information content (PIC) and reproducibility [27–29]. While use of functional molecular markers, such as those developed from expressed sequence tags (EST), directly access to the population diversity of important genes for agriculture, making it easier to link genotype to phenotype. Although, SNPs are the most important DNA markers as they have low levels of recurrent mutations, making them stable in terms of evolution. Therefore, they are the best markers for dissecting the genetic basis of complex characters for analyzing genomic evolution processes [30]. While SNPs can be used as to assess genetic diversity in agricultural species, they are less preferred than SSRs due to their limited information content, biallelic nature, and high cost [19]. A comparative genetic diversity study on sugar beet cultivars using DArT, SNPs, and SSRs showed that SSR markers had the highest success rate due to their highly polymorphic characteristics [31, 32]. Other studies have shown that SSR markers were extremely effective in estimating genetic diversity and association among soybean accessions [16, 28–32].

Since soybeans are a relatively new crop in Pakistan, local breeding programs focus on creating new, competitive cultivars with excellent production and quality with limited attention to understanding the extent of diversity in their working germplasm. Therefore, it is important to assess the genetic diversity of the soybean germplasm from USDA to develop new and improved soybean cultivars for Pakistan. Hence, this study analyzed 96 accessions of cultivated soybean from various geographical regions using 80 genomic SSR and 20 functional EST-SSR markers evaluate the geographic and genetic differences.

## Materials and methods

### Plant material

**Fig 1:**
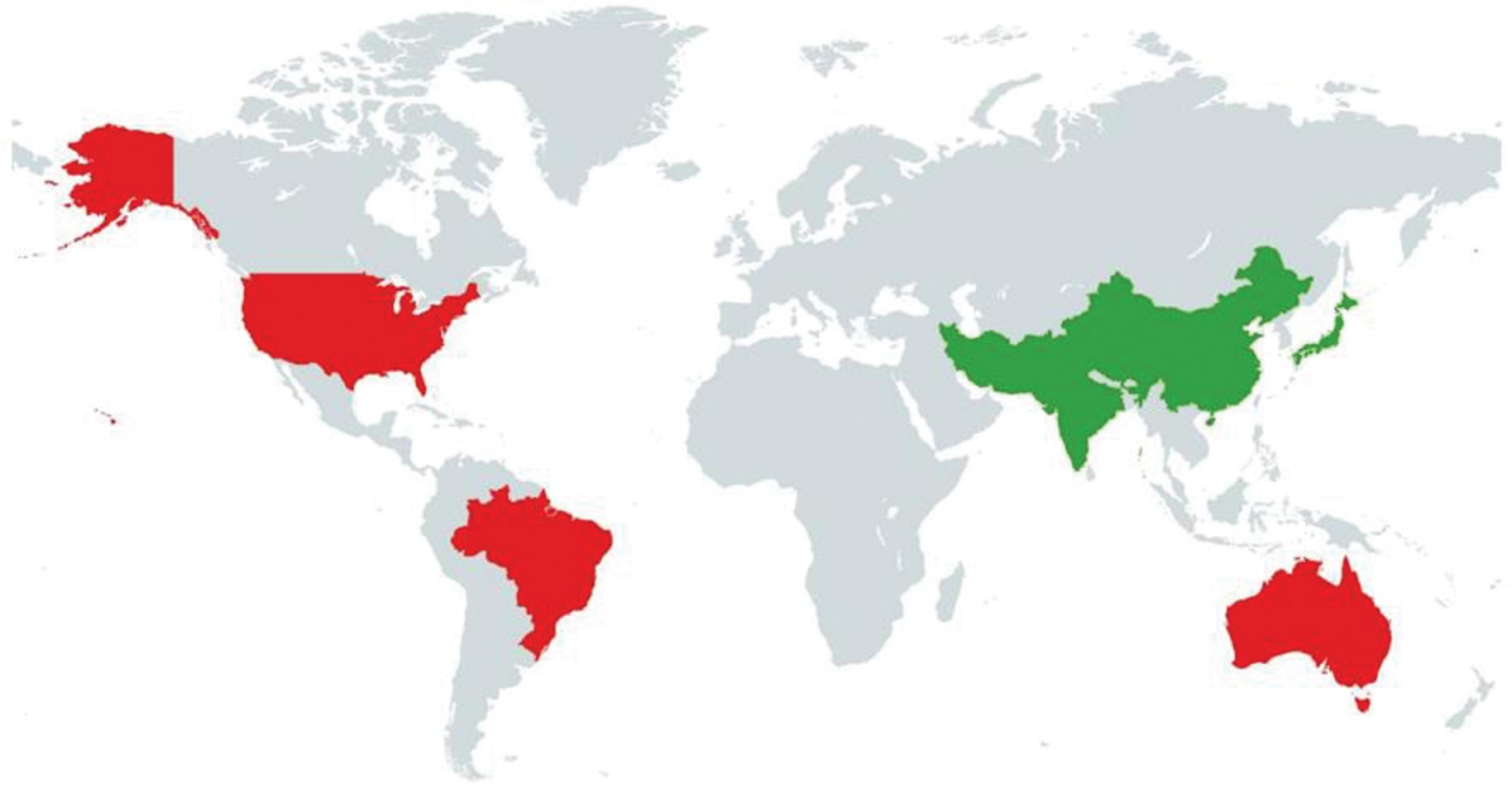
World map representing geographic regions of the 96 accessions used in the study. Created using map chart (mapchart.com).

Ninety-six soybean accessions were collected from various geographical regions and grouped based on origin (Fig 1 and S1 Table). Of these, 59 accessions came from the USA, followed by China (12), Pakistan (8), Brazil (7), India (4), Australia (2), Afghanistan (2), Iran (1), and Japan (1). All accessions were grown in pots in a controlled environment to collect leaf samples.

### Genotyping

Fresh young leaves were used for genomic DNA extraction following the Cetyltrimethyl ammonium bromide (CTAB) method described by Doyle and Doyle [33]. Eighty genomic SSR markers and 20 functional EST-SSR markers distributed uniformly across the soybean genome were selected from the literature (S2 Table) and used to check genetic diversity among the soybean accessions. A PCR reaction mixture (15 μl) was prepared, comprising 1.5 mM of 10× buffer, 3.5 mM MgCl_2_, 600 μM dNTPs, 0.6 μM of each forward and reverse primer, and 1 U Taq polymerase with 50–100 ng DNA. The reaction began with initial denaturation at 94°C for 5 min, followed by 95°C for 30 sec, 48–55°C for 1 min, 72°C for 1 min, and a final extension at 72°C for 10 min. A gradient thermal cycler (Kyratec Super Cycler) was used to perform the PCR reaction. The PCR products were fractionated in 2.5% agarose gel electrophoresis containing ethidium bromide for staining bands and visualized using a UV Analyzer based on their migration distance relative to Gene ruler 50 bp DNA ladder (Thermo Scientific, 10416014).

### Statistical analysis

Genotypic data obtained from the SSR and EST-SSR markers were scored as 0 or 1 based on the presence or absence of a DNA band in the gel (S1 Fig). The expected heterozygosity (He), observed heterozygosity (Ho), genetic distance between accessions (GD), and Shannon informative index (I) were estimated using POPGENE (v.1.32) software [34]. The PIC, gene diversity, and allele frequency of markers were calculated using Power Marker v.3 [35].

### Diversity analysis and population structure

Phylogenetic analysis was conducted using genotypic data to evaluate the dissimilarity among accessions using the Unweighted Pair Group Method with Arithmetic Mean (UPGMA) method in DarWIN software. The phylip file obtained from DarWIN was used to construct the phylogenetic tree in MEGA6 software [36]. Principal coordinate analysis (PCoA) was conducted using Past 4.0 software to identify the degree of differentiation between accessions [37]. Model-based software STRUCTURE v.2.3.4 with the admixture ancestry model was used to analyze population structure, with correlated allelic frequencies used as parameters for the analysis [38]. The number of iterations for the Burn-in Period and Markov Chain Monte Carlo was set at 10,000. The online platform STRUCTURE HARVESTER was used to obtain Optimum Evanno’s K values [39]. Analysis of molecular variance (AMOVA) was used to assess genotypic variation in the population, with accessions from each country considered a single population. Since each population must contain at least two individuals, the accessions from Japan and Iran were considered one population. AMOVA was performed using GenALEx 6.5 software [40].

## Results

### Marker informativeness and heterozygosity

Of the 100 markers used in this study, 17 EST-SSRs and 79 SSRs were polymorphic, with 262 amplified alleles, ranging from 2 to 5 alleles per locus (average 2.79). Of the 96 polymorphic markers, the five most polymorphic markers produced five alleles, followed by seven, 41, and 43 markers that produced four, three, and two alleles, respectively (Table 1). Sat_304 had the maximum PIC value (0.72), and GMES6776 had the minimum (0.16), with an average of 0.44 for the 96 SSR markers. Twenty-eight markers had PIC values ≥0.50, 64 had PIC values ranging from 0.30–0.49, while four had PIC values <0.30. GMES6776 had the highest major allele frequency (0.89), and Sat_304 had the lowest (0.31), with 54 markers ranging from 0.4–0.6 (average 0.57). The average expected heterozygosity (He) and observed heterozygosity (Ho) were 0.5 and 0.02, respectively. Sat_304 and Satt 373 had the maximum heterozygosity of 0.72. The maximum Shannon’s information index was 1.362, while the minimum was 0.356, with the average 0.794 (Table 1).

**Table 1:** Genetic diversity parameters for 96 SSR and EST-SSR markers.

Marker	Major allele frequency	PIC	Allele No	Obs_Het	Ext_Het*	Nei **	I
Sat_304	0.3125	0.72	5	0.0729	0.7324	0.728	1.3627
Satt114	0.3542	0.68	4	0	0.7349	0.731	1.3491
Satt706	0.3854	0.66	4	0	0.7227	0.719	1.3264
Satt510	0.3854	0.66	4	0	0.7171	0.713	1.3089
Satt373	0.3542	0.65	5	0.0729	0.6732	0.669	1.1466
Satt277	0.4792	0.64	4	0.0417	0.6725	0.669	1.2401
Satt336	0.4792	0.60	4	0.0208	0.6507	0.647	1.1593
Satt600	0.413	0.60	4	0.1848	0.5982	0.594	0.9784
Satt180	0.3854	0.60	3	0.0312	0.6551	0.651	1.0748
BE475343	0.4375	0.60	3	0.0312	0.6482	0.644	1.0672
Satt070	0.3958	0.59	5	0.0312	0.6576	0.654	1.121
Satt571	0.3646	0.59	3	0.0104	0.6551	0.661	1.0908
AF162283	0.4792	0.59	3	0.0417	0.6283	0.625	1.0397
Sat_424	0.5	0.56	3	0.0938	0.5955	0.592	0.9728
Satt385	0.5	0.56	3	0.0521	0.5934	0.590	0.9753
Satt150	0.4375	0.56	3	0	0.6431	0.639	1.0561
GMES6352	0.4167	0.56	3	0	0.642	0.638	1.0529
Satt389	0.4688	0.55	5	0.0938	0.5753	0.572	0.9797
Satt434	0.4271	0.55	3	0.0208	0.6169	0.613	1.0072
Satt718	0.5417	0.55	5	0.0312	0.6017	0.598	1.0457
Satt300	0.4792	0.55	3	0.0521	0.5885	0.585	0.9558
Satt478	0.5104	0.54	3	0.0208	0.6014	0.598	0.9909
Satt243	0.4896	0.53	3	0.0312	0.5929	0.589	0.967
Satt154	0.5521	0.52	3	0	0.5975	0.594	0.9971
Satt173	0.4583	0.52	3	0.0208	0.5901	0.587	0.9541
GMES6346	0.5104	0.51	3	0	0.5988	0.595	0.9816
Satt266	0.5	0.50	3	0.0208	0.5782	0.575	0.9333
Satt431	0.4688	0.49	3	0.0312	0.5591	0.556	0.8831
Sat001	0.5699	0.49	4	0.0108	0.5621	0.559	0.9277
GMES1792	0.4896	0.49	3	0.0104	0.5732	0.570	0.9193
Satt687	0.4896	0.46	3	0	0.5665	0.563	0.9023
Satt038	0.4896	0.46	3	0	0.5665	0.563	0.9023
Satt636	0.6042	0.45	3	0.0312	0.5081	0.505	0.816
Satt641	0.5208	0.45	3	0	0.5541	0.551	0.8778
Satt453	0.5417	0.44	3	0.0417	0.5037	0.501	0.7345
Sat-272	0.5313	0.44	2	0.0521	0.496	0.493	0.6866
Satt516	0.5521	0.43	2	0.0521	0.4903	0.487	0.6809
Satt521	0.5	0.43	3	0.0417	0.5067	0.504	0.721
Sat-137	0.5	0.43	3	0.014	0.5317	0.528	0.8097
Satt588	0.6563	0.43	3	0.0104	0.4891	0.486	0.8164
Satt414	0.6354	0.42	3	0.0104	0.495	0.492	0.8018
Satt267	0.5	0.41	3	0	0.5321	0.529	0.81
Satt302	0.5	0.41	2	0.0312	0.5021	0.499	0.6927
Satt187	0.5417	0.41	2	0.0312	0.496	0.493	0.6866
Satt316	0.5729	0.41	2	0.0312	0.4869	0.484	0.6774
GMES6336	0.6979	0.40	3	0	0.4631	0.460	0.7997
Satt539	0.5851	0.40	3	0.0319	0.4821	0.479	0.6726
Satt614	0.625	0.40	3	0	0.4991	0.496	0.7924
Satt022	0.5	0.40	3	0	0.5227	0.52	0.7797
GMES0002	0.5208	0.40	2	0.0208	0.5007	0.498	0.6912
Satt102	0.6042	0.40	2	0.0312	0.4738	0.471	0.6642
Satt194	0.5417	0.40	2	0.0208	0.4972	0.494	0.6877
Satt273	0.5417	0.40	2	0.0208	0.4972	0.494	0.6877
Satt285	0.5417	0.40	2	0.0208	0.4972	0.494	0.6877
Sat-330	0.5521	0.40	2	0.0208	0.4948	0.492	0.6853
Satt565	0.7188	0.39	3	0.0208	0.426	0.423	0.7418
Satt322	0.625	0.39	2	0.0312	0.4629	0.460	0.6531
Sct-189	0.6563	0.39	2	0.0417	0.4396	0.437	0.629
Satt146	0.6667	0.39	3	0	0.4712	0.468	0.7621
Satt564	0.6042	0.39	3	0	0.4965	0.493	0.7529
GMES6391	0.5	0.39	2	0.0104	0.5026	0.499	0.6931
Satt665	0.5313	0.38	2	0.0104	0.4999	0.497	0.6905
Satt634	0.6146	0.38	2	0.0208	0.4712	0.468	0.6616
Satt558	0.6354	0.38	2	0.0208	0.4599	0.457	0.65
Sct_010	0.5938	0.38	2	0.0104	0.4829	0.480	0.6734
Satt342	0.6146	0.37	2	0.0104	0.4738	0.471	0.6642
Satt635	0.5208	0.37	2	0	0.5017	0.499	0.6923
Sctt_008	0.6563	0.37	3	0	0.4671	0.464	0.7221
Satt487	0.5417	0.37	2	0	0.4991	0.496	0.6897
Satt519	0.625	0.37	3	0	0.4788	0.476	0.7092
Satt409	0.6875	0.37	2	0.0312	0.4197	0.417	0.6082
Satt650	0.5625	0.37	2	0	0.4948	0.492	0.6853
Satt286	0.6667	0.37	2	0.0208	0.4396	0.437	0.629
GMES0124	0.5729	0.36	2	0	0.4919	0.489	0.6825
Satt077	0.5833	0.36	2	0	0.4887	0.486	0.6792
Satt334	0.6042	0.36	2	0	0.4808	0.478	0.6713
GMES1173	0.6146	0.36	2	0	0.4762	0.473	0.6667
Satt236	0.6354	0.35	2	0	0.4658	0.463	0.656
Satt390	0.6354	0.35	2	0	0.4658	0.463	0.656
Satt045	0.6354	0.35	2	0	0.4658	0.463	0.656
GMES5332	0.6667	0.34	2	0	0.4468	0.444	0.6365
aw310961	0.75	0.34	2	0.0417	0.3551	0.353	0.5383
GMES2561	0.6947	0.33	3	0	0.4264	0.424	0.6153
Satt538	0.6979	0.33	2	0	0.4239	0.421	0.6126
GMES0963	0.7083	0.32	2	0	0.4154	0.413	0.6036
GMES0701	0.7083	0.32	2	0	0.4154	0.413	0.6036
Sat-099	0.75	0.32	2	0.0208	0.3663	0.364	0.5506
Sctt_009	0.7396	0.32	2	0.0104	0.3821	0.380	0.568
Satt130	0.7253	0.31	3	0	0.4007	0.398	0.5879
GMES5822	0.7292	0.31	2	0	0.397	0.395	0.5841
GMES0902	0.7579	0.29	3	0	0.3689	0.367	0.5535
Sat_334	0.8021	0.29	2	0.0417	0.293	0.291	0.4669
Satt386	0.8125	0.26	2	0.0104	0.2997	0.298	0.4749
GMES3041	0.8085	0.26	3	0	0.3113	0.309	0.4884
GMES6735	0.8542	0.21	2	0	0.2504	0.249	0.4154
GMES6776	0.8854	0.18	2	0	0.204	0.202	0.356
**Mean**	0.57067	0.43	2.729	0.019	0.511323	0.508	0.7943
**St. Dev**			262	0.0271	0.1024	0.101	0.7943

PIC: polymorphic information index, Obs_Het: observed heterozygosity, Exp_Het: expected heterozygosity, Nei**: Nei genetic distance, I: Shannon index

### Genetic relationship among 96 soybean accessions based on origin

The 96 tested soybean accessions were grouped into eight populations based on origin. As Japan and Iran only had a single accession each, they were grouped in a single population (Pop-8). The mean alleles per locus (Na) and effective alleles (Ne) of all populations were 2.023 and 1.786, respectively, with Pop-1 having the highest values (2.594 and 2.077, respectively). Shannon’s informative index ranged from 0.337–0.771 (average 0.569). The observed heterozygosity ranged from 0.005–0.027 (average 0.013) and expected heterozygosity ranged from 0.242–0.492 (average 0.378) (Table 2).

**Table 2:** Genetic diversity parameters for the eight soybean populations analyzed using SSR and EST-SSR markers.

Populations	N	Na	Ne	I	Ho	He
Popn 1	58.906	2.594	2.077	0.771	0.024	0.492
Popn 2	11.969	2.427	2.019	0.745	0.007	0.479
Popn 3	2.000	1.490	1.481	0.337	0.010	0.242
Popn 4	7.969	2.406	2.023	0.742	0.016	0.47
Popn 5	3.990	2.052	1.829	0.621	0.013	0.416
Popn 6	2.000	1.510	1.506	0.352	0.005	0.254
Popn 7	6.979	2.188	1.837	0.628	0.027	0.407
Popn 8	2.000	1.521	1.517	0.360	0.005	0.259
Mean	11.977	2.023	1.786	0.569	0.013	0.378
SE	0.652	0.026	0.020	0.012	0.002	0.008

Mean number of different alleles (Na), the mean number of effective alleles (Ne), Shannon’s index (I), observed heterozygosity (Ho), and effected heterozygosity (He).

### Genetic distance (Nei’s measure) analysis

The genetic distance among the 96 soybean accessions from nine regions ranged from 0.079–1.232 (S3 Table). PI612157 from Georgia, USA, and PI462312 from India had the greatest genetic distance of 1.232, followed by PI269518C from Pakistan and PI462312 from India with 1.17. These four accessions had the highest degree of genetic differentiation based on genetic distance. PI644047 and PI644054, both from Georgia, USA, had the smallest genetic distance of 0.079.

### Diversity analysis

The genetic diversity of 96 soybean accessions was assessed through following analysis.

#### Phylogenetic analysis

**Fig 2:**
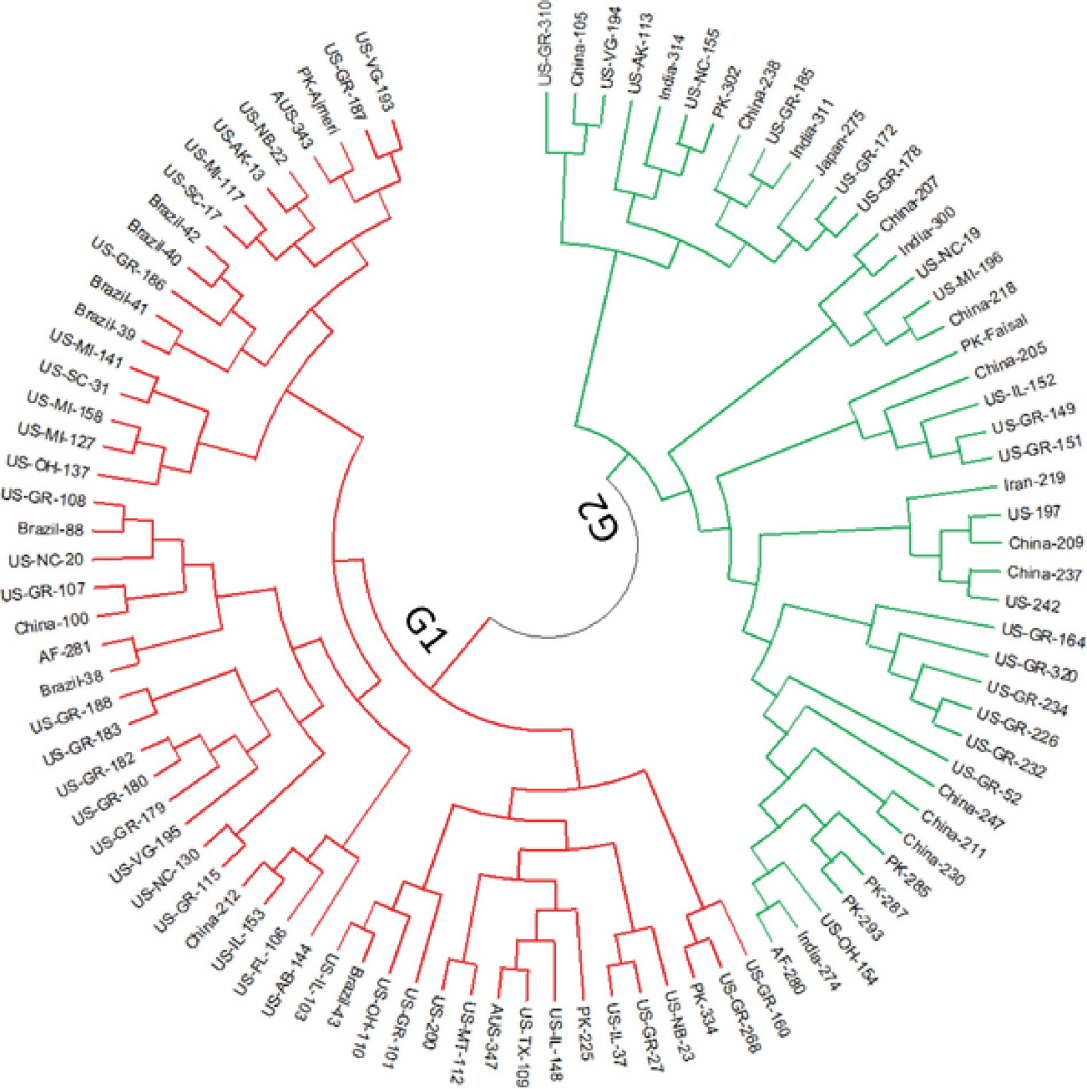
Phylogenetic tree of 96 soybean accessions using the UPGMA method. Accessions in group 1 (G1) are represented with red color while accessions in group 2 (G2) are represented with green color.

**Fig 3:**
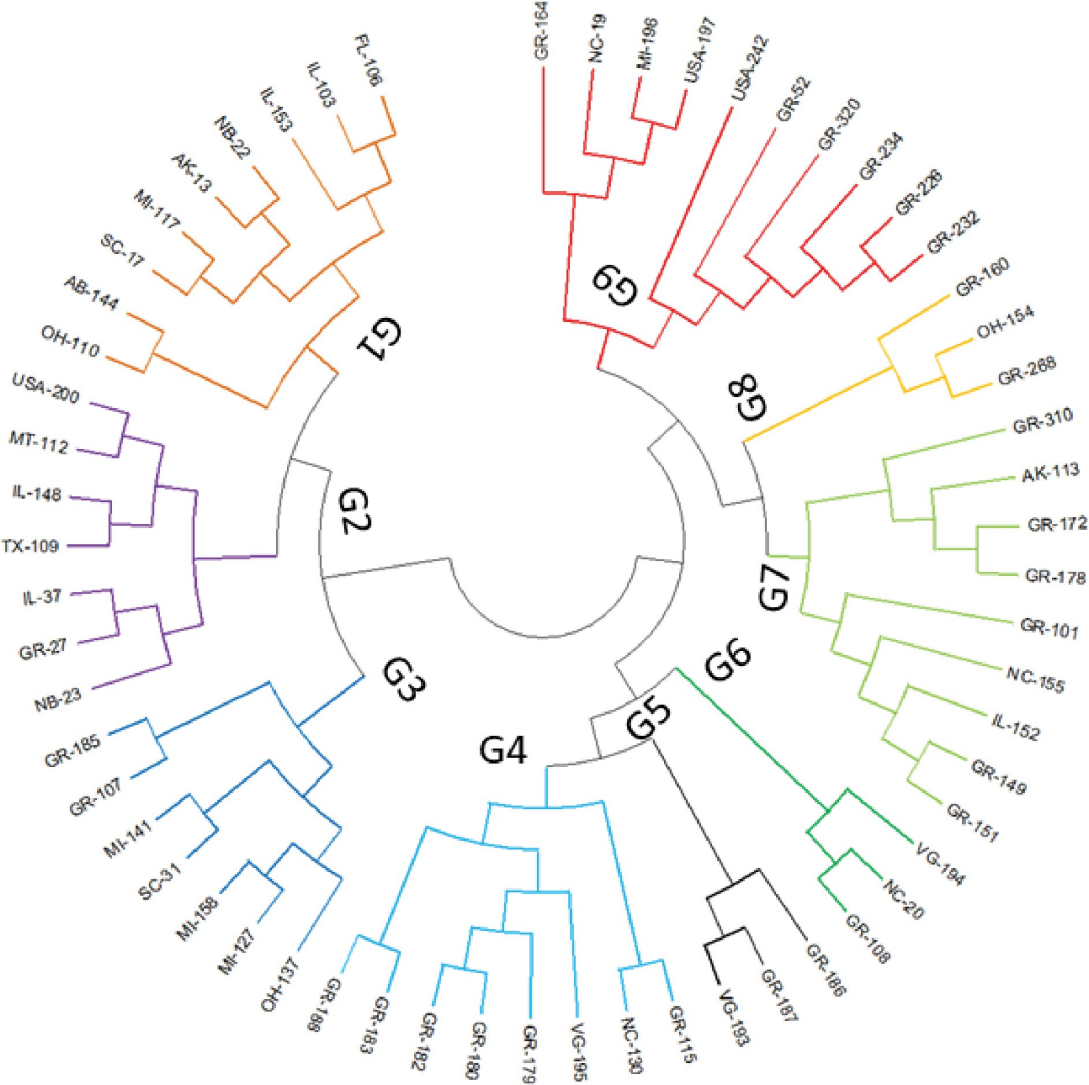
Phylogenetic tree of 59 USA accessions using the UPGMA method.

The phylogenetic analysis identified two major groups and several subgroups (Fig 2). Group-1 (G1) comprised of 53 accessions of which most accessions were from the USA (37) and Brazil (7), while Group-2 (G2) comprised of 43 accessions including accessions from China, Pakistan, India, and Afghanistan. Further G2 contained Pakistani check cv. Faisal while G1 contained cv. Ajmeri. The phylogenetic analysis grouped the 59 USA accessions into nine groups (G1–G9), indicating a high degree of genetic diversity (Fig 3).

#### Principal coordinate analysis

**Fig 4:**
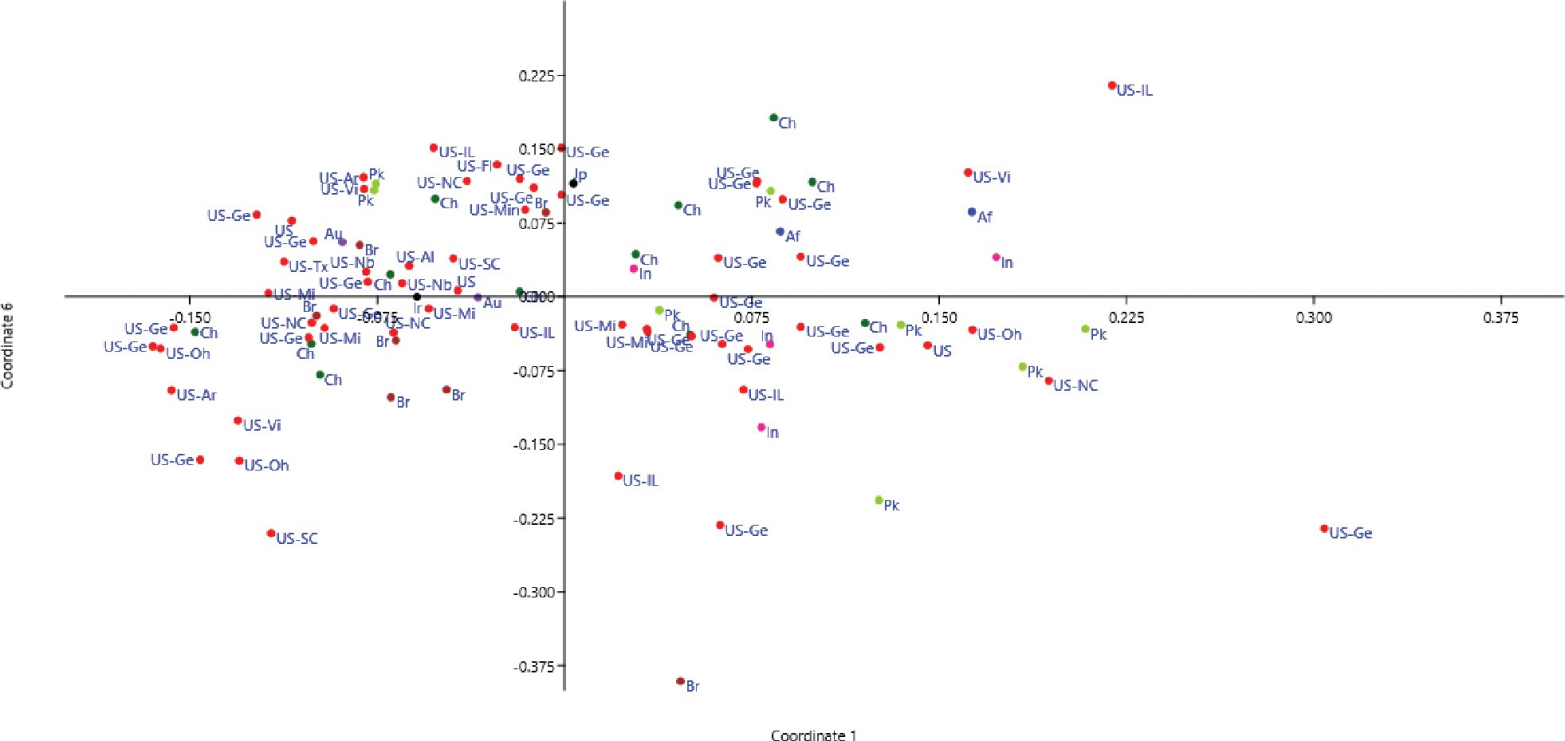
Principal coordinate analysis of 96 soybean accessions using 96 markers to identify variation among accessions based on their country of origin: USA (red), China (brown), Brazil (light green), Pakistan (pink), India (dark green), Iran (black), Afghanistan (blue), Australia (violet), and Japan (gray).

**Fig 5:**
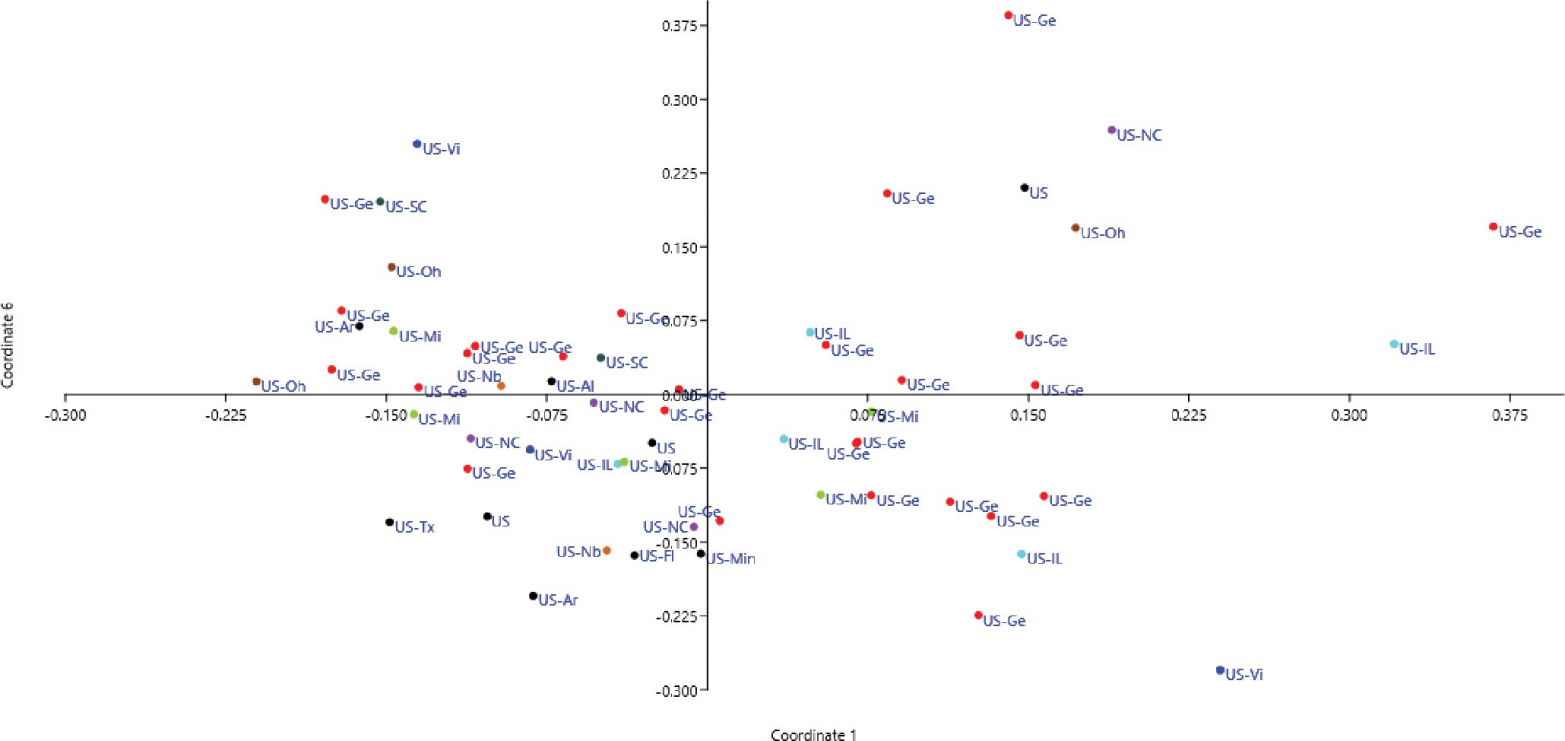
Principal coordinate analysis showing variation between soybean accessions from different states in the USA.

The PCoA showed that all accessions were distributed across the plot, with 45.97% of the total variation explained in the first six coordinates (Fig 4). Based on their grouping, many USA accessions were similar to Brazilian and Australian accessions, while the accessions from India, China, Pakistan, and Afghanistan were similar, with six Chinese and two Pakistani accessions clustered together. A second PCoA of the USA accessions revealed that the accessions were scattered across the plot without any significant clustering. The first six PCoAs explained 51.5% of the total variation (Fig 5).

#### Population STRUCTURE

**Fig 6:**
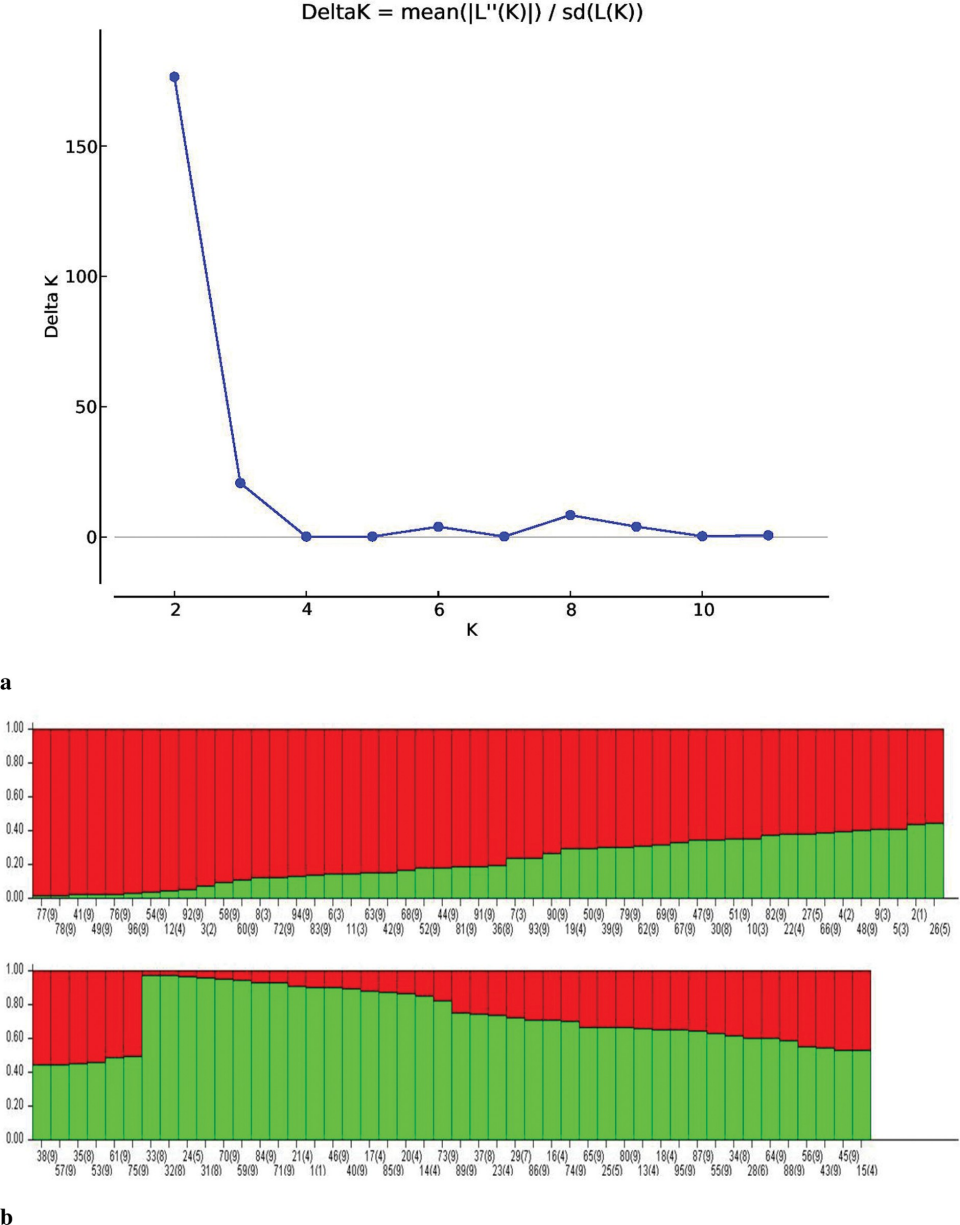
(A) Graph of estimated membership fraction for K = 2. (B) Graphical representation of 96 soybean accessions using 96 markers for K = 2.

**Fig 7:**
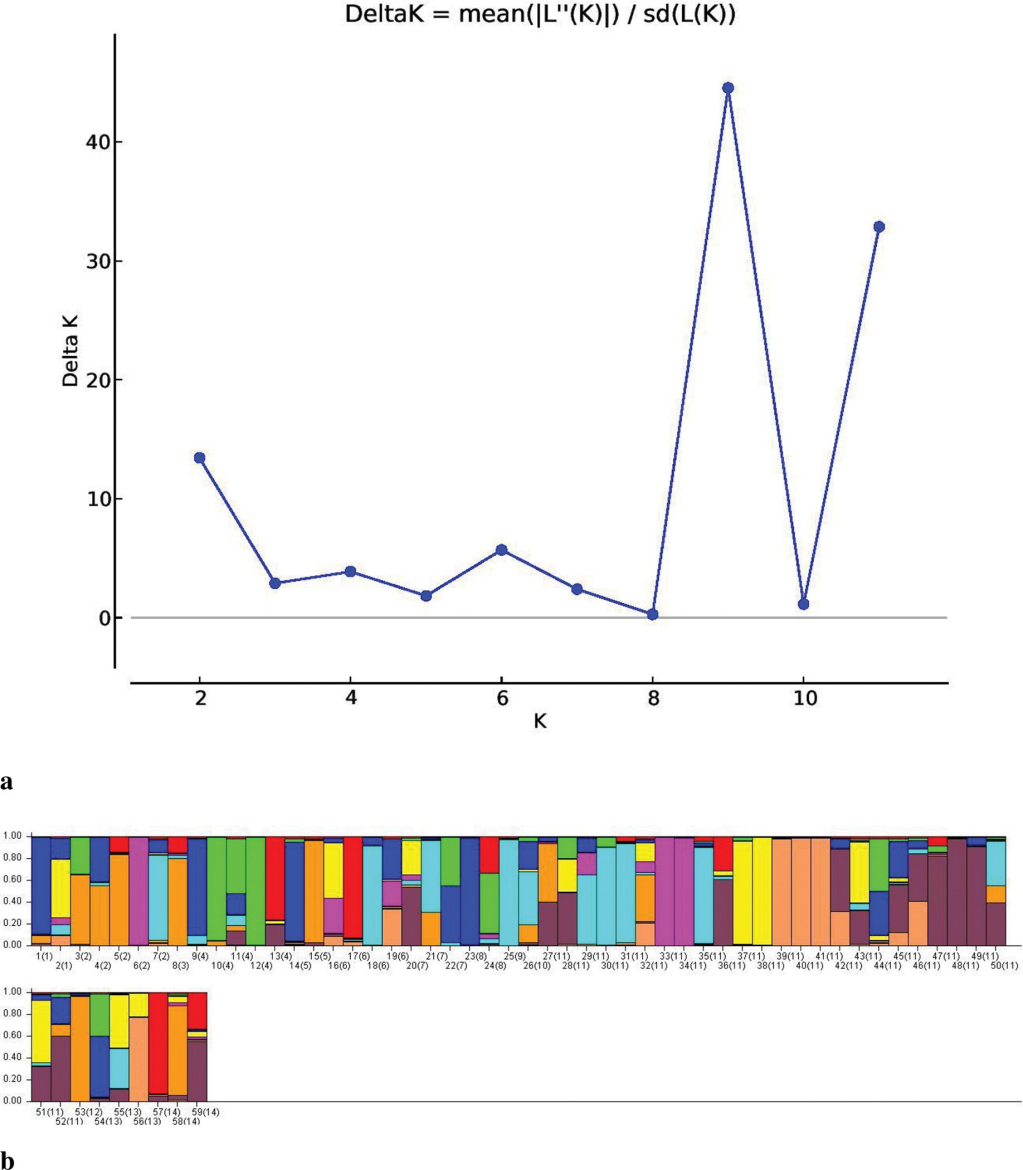
(A) Graph of estimated K value for 59 USA soybean accessions with K = 9. (B) Population structure of 59 USA soybean accessions using SSR and EST-SSR markers.

Population STRUCTURE was used to 1) identify distinct genetic populations, 2) identify migrants and admixed individuals, and 3) assign individuals to populations [41]. The highest peak (K = 2) occurred at ΔK 176.6, indicating that the tested population could be divided into two groups. Two minor peaks, at K = 3 (ΔK = 20.71) and K = 8 (ΔK = 8.38), also occurred (Fig 6A). The accessions with a membership proportion (Q) of 80% or more were considered pure, with the remaining accessions classified as admixture (Fig 6B). At a threshold value of 80%, 42 pure and 54 admixture lines were observed. Of the 42 pure lines, 17 were present in Group-1 (G1 = red), and 25 were present in Group-2 (G2 = green). G1 and G2 contained eight and 19 USA lines, respectively, corresponding to 47% of the pure USA accessions in G1 and 76% of those in G2. At a threshold value of 70%, the number of pure lines increased from 17 to 24 in G1 and 25 to 30 in G2, with the USA lines increasing from 8 to 11 in G1 and 19 to 23 in G2. Three of the 12 Chinese accessions were in G1, with the rest in G2. Five of the eight Pakistani accessions were in G2, with the rest in G1. G1 and G2 each contained two Indian accessions. A similar structure analysis was undertaken for the USA accessions, with an optimum K value of K = 9 obtained (Fig 7A). The results were consistent with the phylogenetic analysis, indicating a high level of differentiation in the USA soybean lines (Fig 7B).

#### AMOVA

The molecular variance observed among individuals within a population was 94% and among populations was only 2% (Table 3). Wright’s F-statistics for the tested markers were F_is_ (0.961) and F_it_ (0.962). The 96 polymorphic markers had a mean fixation index of 0.025, indicating low genetic variation across subpopulations. The rate of gene flow (Nm) was 9.814, indicating a high rate of gene exchange among populations.

**Table 3:** AMOVA of 96 soybean accessions using SSR and EST-SSR markers.

Source	df	SS	MS	Est.Var.	%
Among Populations	7	405.521	57.932	0.621	2%
Among Individuals	88	4210.890	47.851	23.454	94%
Within Individuals	96	90.500	0.943	0.943	4%
Total	191	4706.911		25.018	100%
F-Statistics	Value				
Fst	0.025				
Fis	0.961				
Fit	0.962				
Nm	9.814				

df = degree of freedom, SS = sum of squares, Est.Var = estimated variance, % = percentage variation, Fst = fixation index, Fit = inbreeding coefficient, Fit = overall fixation index

The 59 USA accessions were further grouped based on states and analyzed for AMOVA. The percentage of variation among individuals within the population was 93% and among populations was 2%. Wright’s F-statistics for the tested SSR markers were F_is_ (0.952) and F_it_ (0.953). The SSR markers had a mean fixation index (F_st_) of 0.024, indicating a very low degree of exchange among populations. The rate of gene flow (Nm) was 10.36, indicating a very high rate of gene exchange among populations (Table 4). A pairwise population matrix of accessions was undertaken to check the population distance among populations in three continents: America, Asia, and Australia (Table 5). The results indicated greater genetic diversity among the lines from Asia than continental America and Australia.

**Table 4:** AMOVA of USA accessions from different states using SSR and EST-SSR markers.

Source	df	SS	MS	Est.Var.	%
Among Populations	10	513.862	51.386	0.571	2%
Among Individuals	48	2217.469	46.197	22.531	93%
Within Individuals	59	67.000	1.136	1.136	5%
Total	117	2798.331		24.238	100%
F-Statistics	Value				
Fst	0.024				
Fis	0.952				
Fit	0.953				
Nm	10.359				

df = degree of freedom, SS = sum of squares, Est.Var = estimated variance, % = percentage variation, Fst = fixation index, Fit = inbreeding coefficient, Fit = overall fixation index

**Table 5:** Pairwise population matrix of genetic distance measured across the genotypes from different continents.

Continental America	Asia	Australia	
188.921	199.518	189.371	**Continental America**
199.518	200.706	202.643	**Asia**
189.371	202.643	193.000	**Australia**

## Discussion

Characterizing germplasm and understanding its genetic diversity are prerequisite steps for developing improved crop cultivars [42]. The plant breeders could increase the genetic base of locally adapted cultivars by using their knowledge of genetic diversity. Consequently, genetic diversity estimation has become an important method for locating genetically different parents that possess desirable features is genetic diversity estimate [42]. The identification of genetic diversity and genetic structure of evaluated soybean germplasm using molecular data supported the selection of possible parents based on morpho-biochemical properties, which facilitated long-term breeding and selection operations. In order to reduce the genetic instability of segregating populations, various parents are preferable in soybean crossbreeding [43]. Many studies have been conducted to assess the genetic diversity in legume crops using molecular markers [44, 45]. The polymorphism observed by using these molecular analysis was very high that was very likely due to polymorphic nature of SSRs [46, 47]. Thus molecular markers are reliable source for identifying the various soybean populations.

In present study, 100 uniformly distributed genomic SSR and functional EST-SSR markers were used to explore the genetic diversity among 96 soybean accessions. The results of the phylogenetic analyses, PCoA, population STRUCTURE, and AMOVA indicated high genetic variation among the accessions (Figs 2, 4, and 6; Table 2), with a slightly higher average allelic number per locus (2.88) than an earlier study by Bisen, Khare [48], who reported 2.21 average alleles per locus for 50 SRR markers in 38 soybean accessions. The difference in allele numbers may be due to the different sample sizes, number of markers, and genotypes used [49]. A marker with a PIC value = 0.5 or more indicates the presence of high informativeness [50]. Here, PIC values ranged from 0.18–0.72 (average 0.44), lower than the average 0.47 reported elsewhere [51]. Markers with high PIC values can be used to distinguish soybean accessions. Information on Ho and He suggests the extent of genetic variability in the population [5]. This study had a much lower average Ho (0.019) than average He (0.51), which may be due to the high self-pollinating nature of soybean [52, 53]. The average Shannon’s Index per locus was 0.79, slightly higher than Ullah et al. [54], who reported an average of 0.69 per marker in soybean that shows that diversity observed in present study is slightly higher than previous.

The study population was divided into two major groups, G1 and G2 (Fig 2), with most USA lines in G1 and those from other countries in G2. Žulj Mihaljević [43] also tested 42 SSR markers on 97 European soybean accessions that were separated into two sub-groups based on geographic origin, which also supported the present findings. Geographic distances and genetic variation are highly correlated, which is more likely the result of long-term selection and ecological diversity [55]. These groupings were further supported by PCoA and population STRUCTURE (Figs 2 and 4), suggesting that the USA lines are genetically distinct from lines from other countries and affirming the assumption that USA lines are somewhat distinct from lines from other continents. Similar results obtained by structure and PCoA indicates that two separate gene pools were the primary source of two sub populations [45]. However, some Brazilian lines were also present in G1, which may be due to their close geographical locations or free movement of the germplasm in this region. In addition, most Pakistani lines clustered with the USA lines in G1, possibly due to their similar origins. In an earlier study, Iqbal, Naeem [56] also reported that accessions from USA and Pakistan clustered together. STRUCTURE analysis showed that the ratio of pure USA lines increased when the threshold decreased from 80% to 70% in both groups, in line with the findings of other studies [51, 54, 56]. AMOVA showed a high percentage of variance among individuals within species (94%), supporting the high diversity argument. Similarly, Wen et al. [57] reported 2.70% variation among the population and 97.30% within a subpopulation. The UPGMA, PCoA, population structure, and AMOVA of the 59 USA lines identified a high level of genetic diversity among these accessions (Figs 3, 5, and 7B; Table 3) but also observed genetic similarity among some lines. Other studies support the high degree of variation among the USA lines [58, 59]. The local commercial varieties Faisal soybean and Ajmeri were present in different clusters, indicating that these genotypes were introduced from various origins and assessed throughout the selection process before being made available for commercial cultivation [60]. Appiah-Kubi et al. [61] assessment of the genetic diversity among the soybean genotypes using 20 SSR markers revealed a close link between genotypes and their geographic origin, which is consistent with these findings. Due to the substantial exchange of genetic resources among farming communities, the poor structure of germplasm may be a reflection of the presence of gene flow between the subpopulations [62]. So, by using molecular markers it will be easy for the farmers to assess the suitable genotype for the crossing that may lead to the development of new varieties.

## Conclusion

Frequent use of closely related cultivars reduces the genetic diversity in germplasm and hinders the breeding of new cultivars with improved traits. The soybean accessions investigated in this study are highly diverse, with a medium to high level of genetic variation across geographic regions, Genetic markers can be considered as a useful source to access the genetic diversity which is not easy to achieve through phenotypic diversity thus could be valuable for future breeding programs by increasing the new varieties in already existing gene pool.

## Supporting information

S1 Fig Gel images of PCR product on 2.5% agarose gel (A) Satt150, (B) Satt173, (C) Satt316, (D) Satt373, (E) Satt565, (F) Satt636, (G) Satt706 and (H) Sct189.
(DOCX)

S1 Table List of germplasm used in the study. (XLSX)

S2 Table List of SSR and EST-SSR markers. (XLSX)

S3 Table Genetic distance found between 96 accessions investigated in this study. (XLSX)
