# Supplementary material for: Genetic diversity and population structure analysis in cultivated soybean (Glycine max [L.] Merr.) using SSR and EST-SSR markers
Source: PLoS One. 2023 May 31;18(5):e0286099. doi: 10.1371/journal.pone.0286099 (PMC10231820; doi:10.1371/journal.pone.0286099)
Supplement: S1 Fig — Gel images of PCR product on 2.5% agarose gel (A) Satt150, (B) Satt173, (C) Satt316, (D) Satt373, (E) Satt565, (F) Satt636, (G) Satt706 and (H) Sct189. (DOCX) [file pone.0286099.s001.docx]

A


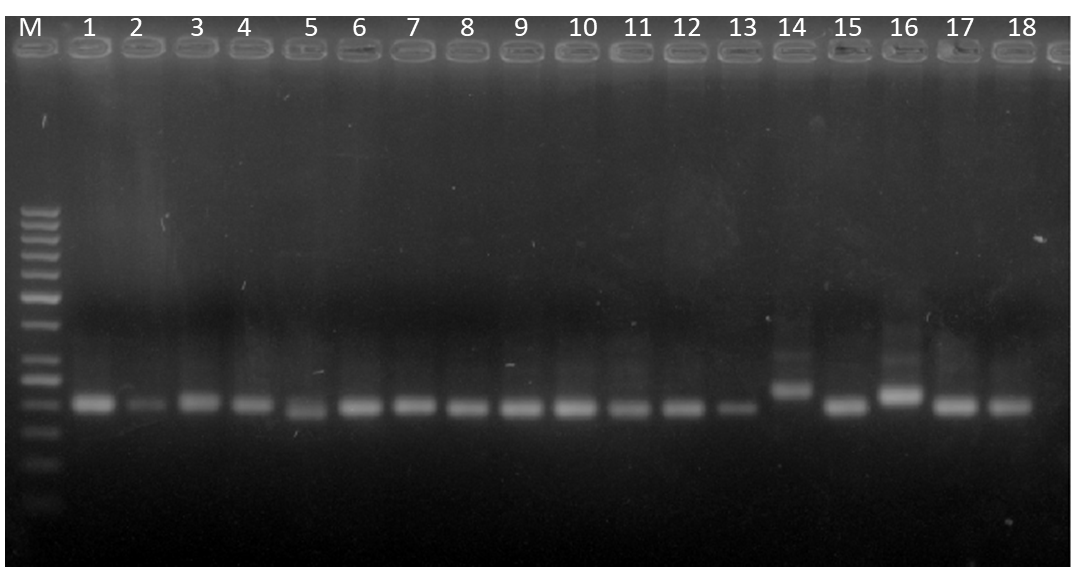


B


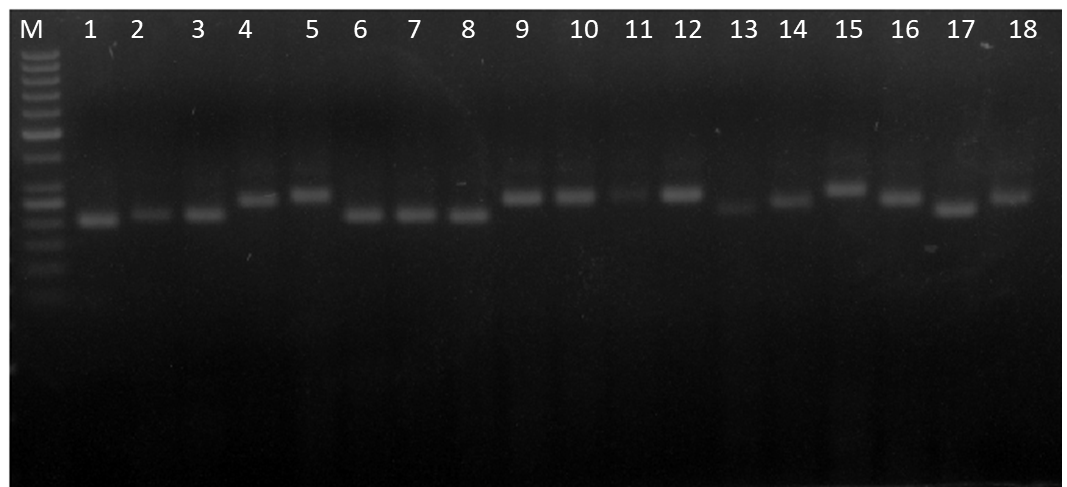


C


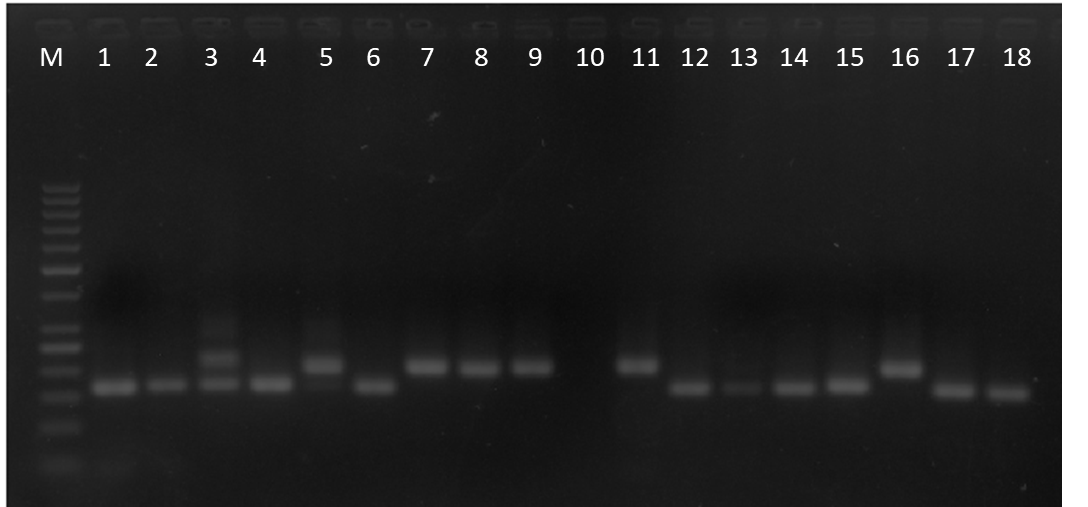


D


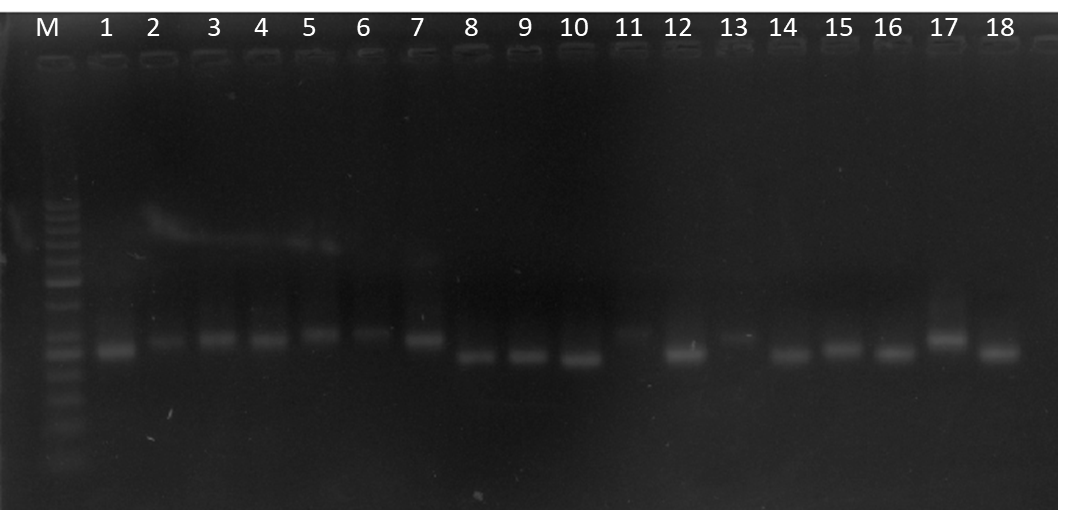


E


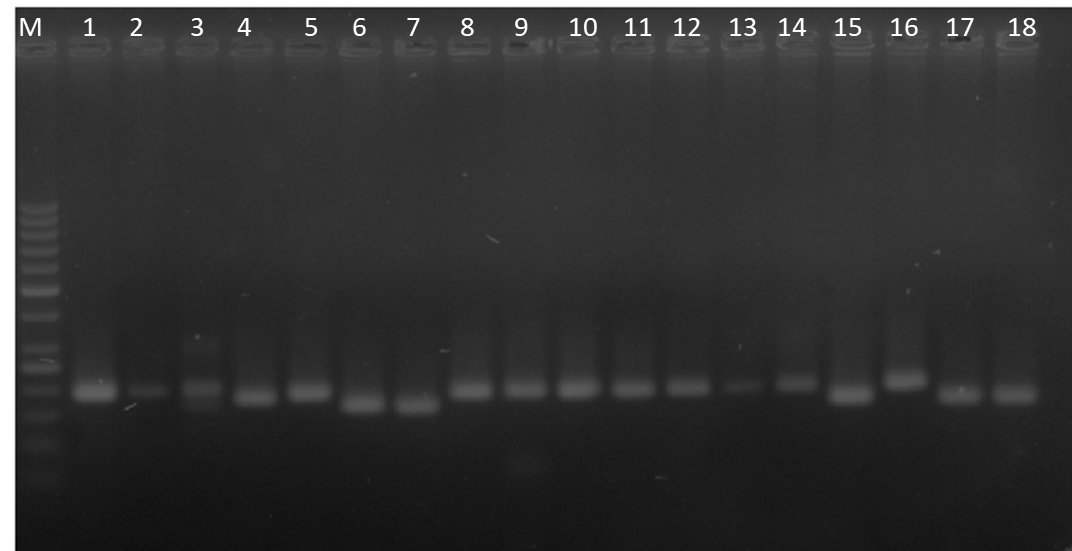


F


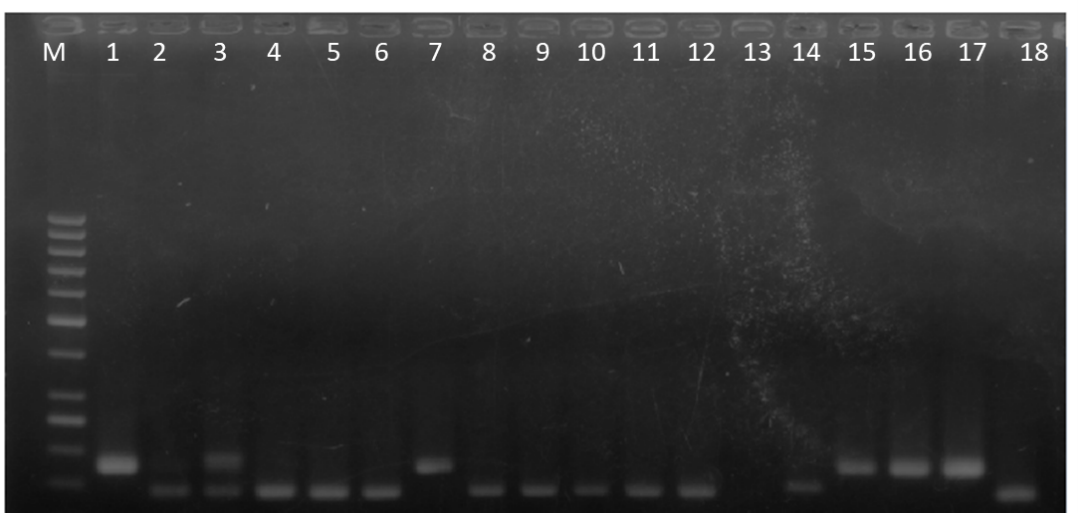


G


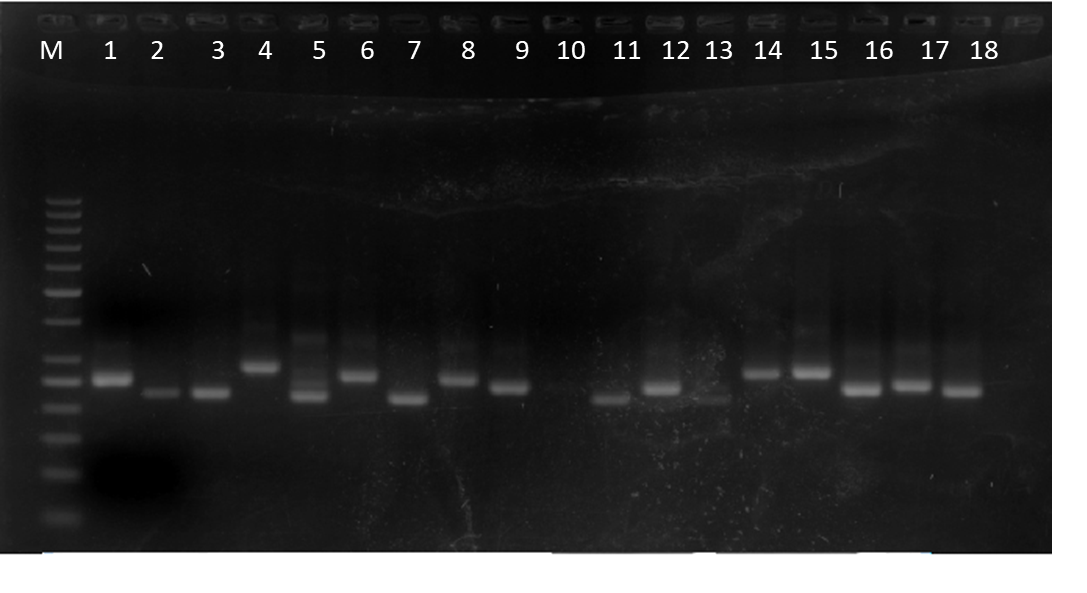


H


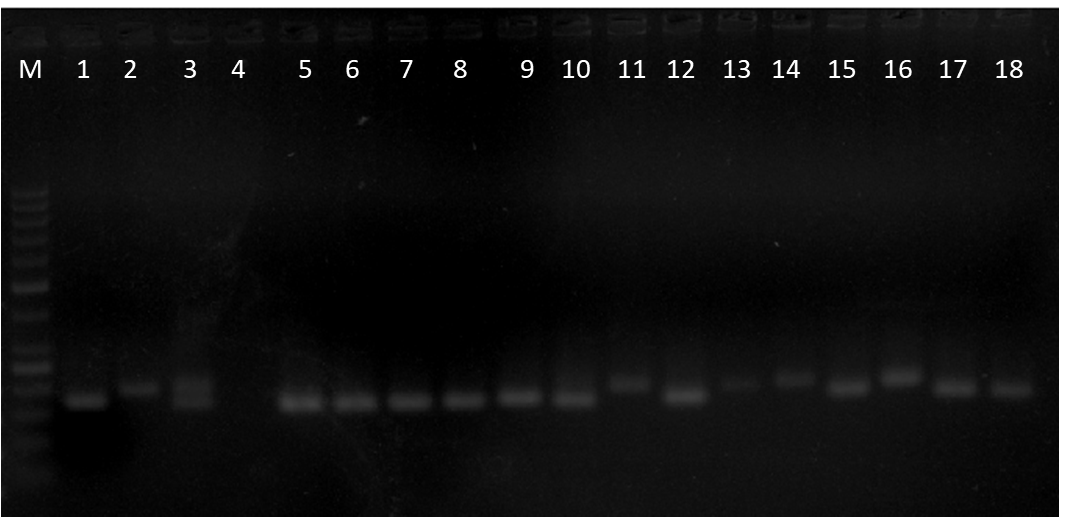


Fig 1. Gel images of PCR product on 2.5% agarose gel (A) Satt150, (B) Satt173, (C) Satt316, (D) Satt373, (E) Satt565, (F) Satt636, (G) Satt706 and (H) Sct189
